# Neuromuscular Performance Changes in Response to the Australian Special Forces Selection Course

**DOI:** 10.1093/milmed/usaf541

**Published:** 2026-04-17

**Authors:** Angela Uphill, Kristina L Kendall, Stuart Guppy, Michael Vacher, Simon Parker, Hannah Brown, Brendan Ashmore, Travis Zomer, G Gregory Haff

**Affiliations:** School of Medical and Health Sciences, Edith Cowan University, Joondalup, WA 6027, Australia; Australian Defence Force, Paddington, NSW 2021, Australia; School of Medical and Health Sciences, Edith Cowan University, Joondalup, WA 6027, Australia; School of Medical and Health Sciences, Edith Cowan University, Joondalup, WA 6027, Australia; Australian eHealth Research Centre, CSIRO Health and Biosecurity, Kensington, WA 6151, Australia; Australian Defence Force, Paddington, NSW 2021, Australia; School of Medical and Health Sciences, Edith Cowan University, Joondalup, WA 6027, Australia; Australian Defence Force, Paddington, NSW 2021, Australia; Australian Defence Force, Paddington, NSW 2021, Australia; Australian Defence Force, Paddington, NSW 2021, Australia; School of Medical and Health Sciences, Edith Cowan University, Joondalup, WA 6027, Australia

## Abstract

**Introduction:**

Strenuous military activities, such as Special Forces (SF) selection courses, are known to involve high physical loads in the presence of food and sleep deprivation, which can have detrimental impacts on physical performance. Understanding the magnitude of performance deficit and the time required for recovery associated with these events, is important for injury prevention and maintaining operational readiness. The purpose of this study was to investigate the changes in physical performance in response to the Australian SF Selection Course (SF-SC) and to monitor recovery throughout 8 weeks following the course.

**Materials and Methods:**

Ninety-three healthy male participants (age: 28 ± 4 years) were recruited; follow-up data were available from those who successfully completed the SF-SC (*n* = 21). The isometric mid-thigh pull (IMTP), countermovement jump (CMJ), serum hormone concentrations (total testosterone, free testosterone, cortisol, sex hormone binding globulin [SHBG], testosterone:cortisol ratio [T:C]), and body composition (body mass, lean mass) were assessed before the course, immediately post-course, and at 1, 3, 5, and 8 weeks post-course. This study employed Wilcoxon rank tests for pairwise comparisons between baseline and immediate post-course measures, as well as baseline measures with subsequent time points (weeks 1, 3, 5, and 8) to assess recovery time. Additionally, linear mixed effects models (LMEs) were used to analyze the effects of body composition and hormones on physical performance.

**Results:**

IMTP peak force was reduced by 16 ± 11% (*P* < .01, ES = 0.83, 95% CI [0.66-0.88]), and peak force relative to lean mass was reduced by 13 ± 13% (*P* < .05, ES = 0.76, 95% CI [0.54-0.88]) in response to the SF-SC. No other measured IMTP variable demonstrated statistically significant change in response to the SF-SC, and all measured IMTP variables had returned to baseline levels at 1 week post-course. Jump height was reduced by 28 ± 9% (*P* < .001, ES = 0.88, 95% CI [0.88-0.88]) in response to the SF-SC. CMJ peak power and mean power were reduced by 30 ± 5.6% (*P* < .001, ES = 0.88, 95% CI [0.88-0.88]), and 29 ± 7.5% (*P* < .001, ES = 0.88, 95% CI [0.88-0.88]), respectively. Concentric peak force was reduced by 26 ± 10% (*P* < .001, ES = 0.88, 95% CI [0.88-0.88]), breaking peak force by 28 ± 26.3% (*P* < .01, ES = 0.86, 95% CI [0.76-0.88], and flight time:contraction time (F:T) by 26 ± 10.8% (*P* < .001, ES = 0.88, 95% CI [0.88-0.88]). CMJ variables took between 1 and 3 weeks to recover to baseline. LMEs demonstrated that total testosterone, free testosterone, and the T:C ratio all had a significant effect on CMJ jump height and peak power and IMTP peak force and peak rate of force development (RFD). Body mass and lean mass exhibited significant positive effects on peak power and peak force, while they did not influence jump height or peak RFD.

**Conclusions:**

The SF-SC resulted in large decreases in strength and power, that took 3 weeks to fully recover. The results of this study can be utilized to inform training and recovery intervention following highly strenuous military training.

## INTRODUCTION

Military personnel undergo rigorous training to prepare for the demands of their roles, both domestically and in preparation for overseas operations. Training programs are often associated with multifactor stress, including high intensity physical activity, heavy load carriage, inadequate nutritional intake, cognitive and psychological stress, and deliberate sleep deprivation.^1–3^ Exemplifying highly stressful military training events are Special Forces (SF) selection courses which are used to select soldiers who possess the desired attributes to work within elite SF units. These courses are designed to assess potential candidates’ physical and mental capacity, as well as resilience for operating under extreme and unpredictable conditions.^4^^,^^5^ The combined stressors associated with military training, such as SF selection courses, negatively impact physical performance capacity, with ∼15%-30% decreases observed in strength, power, muscular endurance, agility, and aerobic capacity.^3^^,^^5–7^

Understanding the resultant decrements in physical performance following arduous courses is important, as suboptimal strength and power-generating capacities have been linked to a 2 to-3-fold increase in musculoskeletal injury risk, which can subsequently impair operational readiness.^5^^,^^8–10^ Furthermore, strength and power have been associated with performance of operationally relevant tasks, such as casualty evacuation (*r* = −0.64, *P* < .01) and susceptibility to enemy fire (*r* = −0.84, *P* < .001), while changes in lower body strength were significantly associated with changes in shooting performance (*r* = 0.49, *P* < .01) following military field training.^11–13^ Consequently, training activities that induce reductions in strength or power-generating capacity may inadvertently increase injury risk and/or compromise operational effectiveness. Therefore, it is imperative to quantify the extent of decline in physical performance capacities and to understand the time course of recovery following arduous training events. This knowledge is vital for developing and optimizing recovery strategies, as well as ensuring the sustained readiness of military personnel.

This is the second of 2 articles from research investigating the physiological and performance consequences of the 2021 Australian SF Selection Course (SF-SC). Previously, we have reported a high energy expenditure (7680 ± 1095 kcal^.^d^−1^) and negative energy balance (3823 ± 1256 kcal^.^d^−1^) throughout the SF-SC, which was associated with substantial hormonal disturbance, and losses of body mass, fat mass, and lean mass.^14^ This second article reports on the associated changes in physical performance in response to the course.

The primary purpose of this study was to investigate the impact of the SF-SC on muscular strength and power, and the time course of recovery of these factors throughout 8 weeks following the SF-SC. The secondary purpose was to investigate the influence of body composition and hormone concentrations on strength and power generating capacities throughout the SF-SC and recovery period.

## MATERIALS AND METHODS

### Participants

Ninety three healthy male participants (means ± SD; age: 28 ± 4 years, years of service: 7 ± 3 years, height: 1.81 ± 0.07 m, body mass: 85.1 ± 8.1 kg, estimated body fat: 13.7 ± 2.3%, and lean mass: 71.3 ± 6.3 kg) volunteered to participate in this study after providing written informed consent. All participants were current serving members of the Australian Defence Force who had been approved to participate in the 2021 SF-SC. Data were collected from all participants at baseline; however, follow-up data could only be collected from those who successfully completed the SF-SC. This study was approved by the Department of Defence and Veterans Affairs Human Research Ethics Committee (304-20) and Edith Cowan University Human Research Ethics Committee (2021-02253).

### Experimental Design

The details of the SF-SC have been described in detail elsewhere.^14^ In brief, the SF-SC lasted 19 days, consisting of sustained strenuous physical activity, heavy load carriage and cognitive and psychological stress in the presence of food and sleep deprivation. Assessment of physical performance, anabolic and catabolic hormones and body composition were conducted at baseline for all participants, while follow-up assessments were repeated immediately post-course, and at 1 week, 3 weeks, 5 weeks, and 8 weeks post-course, for participants who successfully completed the SF-SC. Immediate post-course measures were collected as soon as the course finished (i.e., blood), and 2 days following completion of the course (body composition and physical performance testing). Dual X-ray absorptiometry (DXA) and blood draws were always conducted between 0600 and 0900, after an 8 hour overnight fast. Physical performance testing was always conducted in a non-fasted state between 1200 and 1500.

### Physical Performance

Physical performance tests were conducted following a standardized warmup. Each warmup was directed by physical performance staff and consisted of dynamic stretching, jogging, skipping, hopping, and submaximal jumping, and lasted approximately 10 minutes.

### Isometric Mid-Thigh Pull

The Isometric Mid-Thigh Pull (IMTP) was conducted with an adjustable portable IMTP rack (VALD Performance, Brisbane, Australia) combined with 2 force plates that sampled at 1,000 Hz (PS-2141, PASCO Scientific, CA, USA). Participants were coached into the stance position corresponding with the second pull of the clean,^15^ with knee and hip angles (knee = 125–145°, hip = 140-150°) confirmed by handheld goniometry. The bar height was recorded at baseline, and the same height was used for all testing sessions. Participants were secured to the bar with lifting straps and instructed to stand quietly for a few seconds without pretension, to establish a stable force baseline. An IMTP specific warm-up was conducted, consisting of submaximal trials at 50%, 75% and 90% of maximal effort, with 1 minute rest between each warm-up trial. Participants were instructed to pull upward on the bar “*as hard and fast as possible*”. Each trial began with a countdown of “*3, 2, 1, Pull!*”, and strong verbal encouragement was given throughout the whole duration of each 5-second trial. The force-time curve was visually inspected following each trial. Trials were considered invalid and repeated in the event of a difference of >250 N peak force between trials, or if a countermovement was detected before initiation of the pull.^16^ A minimum of 2 trials, separated by 1 minute rest, were conducted during each testing time-point, with additional trials conducted in the event of testing error. Force-time curves were analysed using custom LabVIEW software (Version 14.1, National Instruments, Austin TX, USA).^17^^,^^18^ Unfiltered net force-time curves (i.e., body weight removed) were analysed for peak force, force at 100, 200, and 250 ms, peak rate of force development (RFD) and RFD at time bands 0-100, 0-200, and 0-250 ms.^19^ Peak force was also examined relative to body mass (PF/BM) and lean body mass (PF/LBM).

### Countermovement Jump

Countermovement jump (CMJ) testing was conducted on dual portable force plates (PS-2141; PASCO Scientific, CA, USA) using Capstone software (version 2.3; PASCO Scientific, CA, USA). Participants were instructed to perform a maximal CMJ with hands on hips using a self-selected counter movement depth.^20^ After a minimum 1-second weighing phase, standing as still as possible in a standing tall position, the trial was initiated with a countdown of “*3, 2, 1, Jump!*” A minimum of 3 jumps, separated by 1 minute rest, were conducted during each testing time-point. Trials were discarded if the participant was unable to maintain a stable baseline before and during the countdown, if they missed the plates upon landing, if their arms left their hips, or if they were observed to excessively flex their knees while airborne, with additional jumps conducted as required. Right and left leg vertical ground reaction forces were recorded at 1,000 Hz and exported for analysis as individual and summated force-time curves in a custom Excel spreadsheet (Microsoft Inc, WA, USA). Jump height from take-off velocity, concentric peak force, concentric impulse (Imp), peak power, mean power, eccentric RFD, braking RFD, braking peak force, and the ratio of flight time to contraction time (FT:CT), were analyzed at each time-point.

### Body Composition

Body mass and height were assessed with a digital scale (HD-387, Tanita, WA, Australia) and stadiometer (Seca 222, Seca, Hamburg, Germany), and body composition was assessed using DXA (Hologic, Discovery, Bedford, MA, USA). Whole body scans were conducted by trained personnel to estimate lean mass (kg). This study did not utilize the National Health and Nutrition Examination Survey (NHANES) correction factor.

### Hormones

Venous blood samples were collected from the antecubital vein by trained personnel to analyze total testosterone (TT), free testosterone (FT), sex hormone-binding globulin (SHBG), cortisol (Cort), and the ratio between total testosterone and cortisol (T:C). Samples were rested at room temperature for 15 minutes before being centrifuged at 1,500 *g* for 10 minutes, then refrigerated at 4 °C until being analysed on the day of collection (Randox Evidence Investigator, Randox Laboratories, Crumlin, UK). Intra-assay coefficients of variation (%) were: TT: 5.8; FT: 6.8; SHBG: 7.3; Cort: 6.7; and assay sensitivities were: TT: 0.3 nmol L^−1^; FT: 0.0 pmol L^−1^;SHBG: 2.0 nmol L^−1^; and Cort: 30.0 nmol L^−1^.

### Statistical Analyses

Wilcoxon rank tests were used to perform pairwise comparisons between baseline (pre-course) measures and those taken immediately after the course. This test was chosen because of the limited sample size preventing surety that the assumption of normality had been met. The same method was used to compare baseline measures with each subsequent time point post-course (weeks 1, 3, 5, and 8) to estimate recovery time. Wilcoxon effect sizes were also calculated and interpreted using Cohen’s (1988) thresholds, where ES = 0.10, 0.30, and 0.50 represent small, medium, and large effects, respectively.^21^^,^^22^ We used linear mixed effects models (LMEs) to examine the possible effect of body composition and hormones on physical performance. The data included measurements obtained from baseline through to 8 weeks post SF-SC. To determine the optimal model fit, we employed a stepwise variable selection approach guided by Akaike’s information criterion (AIC).^23^ The fixed effects of the model included the participants’ age at baseline (in years), collection times (in days), body composition, and hormones. Additionally, we incorporated a random intercept for each participant to accommodate both within-subject and between-subject variations. To address multicollinearity, separate models were constructed to examine the effects of the different body composition (body mass and lean mass) and hormone measures (TT, FT, and T:C ratio) investigated. All continuous variables were scaled before model fitting to standardize their ranges, ensuring comparability and stability in estimation while preventing issues due to differing units or magnitudes. The resulting regression estimates (β) can be interpreted as standardized effect magnitudes, indicating change in the dependent variable per one unit change in each predictor. Degrees of freedom for all models were computed using the Satterthwaite approximation. All analyses were performed in *R* (R version 4.2.2, http://www.r-project.org) and the lme4 package (version 1.1.33).^24^ Statistical significance was set at *P* < .05.

## RESULTS

Of the 93 participants who started the course, 24 finished, and of these, 20 were selected to continue into SF qualification training. The results presented in the following sections represent only those participants for whom post-course data were available. The number of participants available for follow up varied per time point due to conflicting work-related and personal responsibilities, with maximum *n* = 18 for physical performance variables, *n* = 21 for serum hormones, and *n* = 20 for body composition (**Tables 1-3**).

**Table 1.** Changes in Physical Performance in Response to the Special Forces Selection Course

	Baseline	Immediate post	Post (1 week)	Post (3 week)	Post (5 week)	Post (8 week)
	*n* = 18	*n* = 18	*n* = 14	*n* = 11	*n* = 14	*n* = 13
**IMTP**						
PF/BM (N^.^kg^−1^)	32.6 (4.35)	30.2 (3.94)	29.9 (3.69)	30.7 (4.03)	28.7 (3.79)	29.7 (4.21)
PF/LBM (N^.^kg^−1^)	37.9 (5.31)	32.9 (4.21) *	33.2 (4.12)	35.3 (4.56)	32.2 (4.30)	33.3 (4.53)
Force 100 ms (N)	364 (284)	266 (173)	271 (190)	386 (327)	394 (290)	388 (275)
Force 200 ms (N)	1,040 (452)	721 (457)	781 (441)	944 (692)	1,010 (495)	1,060 (609)
Force 250 ms (N)	1,390 (452)	928 (482)	1,070 (518)	1,150 (685)	1,280 (531)	1,260 (660)
Peak RFD (N^.^s^−1^)	11,300 (3760)	8,920 (3900)	10,300 (3970)	12,300 (6060)	1,2500 (5350)	11,700 (3600)
RFD100 (N^.^s^−1^)	3,300 (2970)	2,350 (1890)	2,640 (1910)	4,080 (3700)	3,820 (2950)	3,910 (2690)
RFD200 (N^.^s^−1^)	5,040 (2350)	3,450 (2380)	3,870 (2220)	4,830 (3700)	4,970 (2460)	5,330 (3020)
RFD250 (N^.^s^−1^)	5,430 (1890)	3,590 (2010)	4,240 (2090)	4,670 (2920)	5,060 (2080)	5,060 (2610)
**CMJ**						
Jump height (m)	0.34 (0.04)	0.24 (0.03) ***	0.24 (0.04) **	0.29 (0.04)	0.31 (0.05)	0.33 (0.04)
Concentric PF (N)	1,260 (268)	917 (174) ***	1,100 (271)	1,210 (259)	1,160 (199)	1,290 (254)
Concentric imp (N^.^s)	218 (15.7)	171 (18.1) ***	186 (24.3) **	210 (18.5)	209 (17.3)	217 (22.1)
Mean power (W)	2,420 (294)	1,720 (215) ***	1,930 (308) **	2,260 (285)	2,240 (230) *	2,440 (295)
Eccentric RFD (N^.^s^−1^)	5,300 (1690)	3,560 (859) **	4640 (1480)	5,420 (1680)	4,990 (1240)	5,700 (1730)
Braking RFD (N^.^s^−1^)	6,570 (2850)	4370 (1460) *	5,680 (2630)	6,830 (2970)	5,870 (1860)	7,030 (2600)
Breaking PF (N)	1,080 (216)	806 (142) **	940 (178)	1,070 (226)	1,010 (168)	1,110 (218)
FT:CT (s)	0.61 (0.08)	0.46 (0.04) ***	0.52 (0.07)	0.57 (0.07)	0.60 (0.07)	0.64 (0.08)

Data are presented as mean (SD). *N* varies at each time point due to participant availability. Abbreviations: FT:CT, flight time:contraction time ratio; Imp, impulse; PF, peak force; PF/BM, peak force relative to body mass; PF/LBM, peak force relative to lean body mass; RFD, rate of force development. Statistical differences represent differences compared to baseline from Wilcoxon rank test at level of significance:

* *P* < .05.

** *P* < .01.

*** *P* < .001.

**** *P* < .0001.

**Table 2.** Changes in Body Composition in Response to the Special Forces Selection Course

	Baseline	Immediate Post	Post (1 week)	Post (3 week)	Post (5 week)	Post (8 week)
	**(** *n* = **20)**	**(** *n* = **20)**	**(** *n* = **17)**	**(** *n* = **14)**	**(** *n* = **19)**	**(** *n* = **17)**
Body mass (kg)	83.2 (6.10)	76.4 (6.12) **	82.5 (6.71)	86.3 (4.98)	85.0 (6.60) *	85.1 (7.36)
Lean mass (kg)	69.9 (4.54)	66.9 (4.92) ****	71.1 (5.64)	72.9 (4.39)	71.2 (5.39) *	70.0 (5.82)

Data are presented as mean (SD). *N* varies at each time point due to participant availability. Statistical differences represent differences compared to baseline from Wilcoxon rank test at level of significance:

* *P* < .05.

** *P* < .01.

*** *P* < .001.

**** *P* < .0001.

**Table 3.** Change in Hormones in Response to the Special Forces Selection Course

	Baseline	Immediate Post	Post (1 week)	Post (3 week)	Post (5 week)	Post (8 week)
	*n* = **21**	*n* = **21**	*n* = **16**	*n* = **14**	*n* = **18**	*n* = **15**
**TT (nmol^.^L** ^−^ **^1^)**	18.8 (4.60)	2.67 (2.10) ****	16.3 (5.03)	15.3 (3.67)	14.8 (4.76) *	15.3 (4.40)
**FT (nmol^.^L** ^−^ **^1^)**	354 (59.5)	25.4 (16.6) ***	293 (49.9) **	349 (61.0)	297 (75.6)	350 (79.5)
**Cort (nmol^.^L** ^−^ **^1^)**	449 (115)	742 (120) ****	355 (79.2)	424 (134)	389 (121)	503 (115)
**T:C ratio**	0.0459 (0.0190)	0.0038 (0.0032) ****	0.0488 (0.0200)	0.0407 (0.0190)	0.0429 (0.0234)	0.0329 (0.0138)
**SHBG (nmol^.^L** ^−^ **^1^)**	36.0 (14.1)	78.3 (24.9) ***	38.9 (12.9)	24.4 (8.63)	31.3 (11.9)	24.7 (8.30)

Data are presented as mean (SD). N varies at each time point due to participant availability. Abbreviations: Cort, cortisol; FT, free testosterone; SHBG, sex hormone binding globulin; T:C, testosterone:cortisol ratio; TT, total testosterone. Statistical differences represent differences compared to baseline from Wilcoxon rank test at level of significance:

* *P* < .05.

** *P* < .01.

*** *P* < .001.

**** *P* < .0001.

### Physical Performance

#### Isometric mid-thigh pull (IMTP)

**Figure 1.**
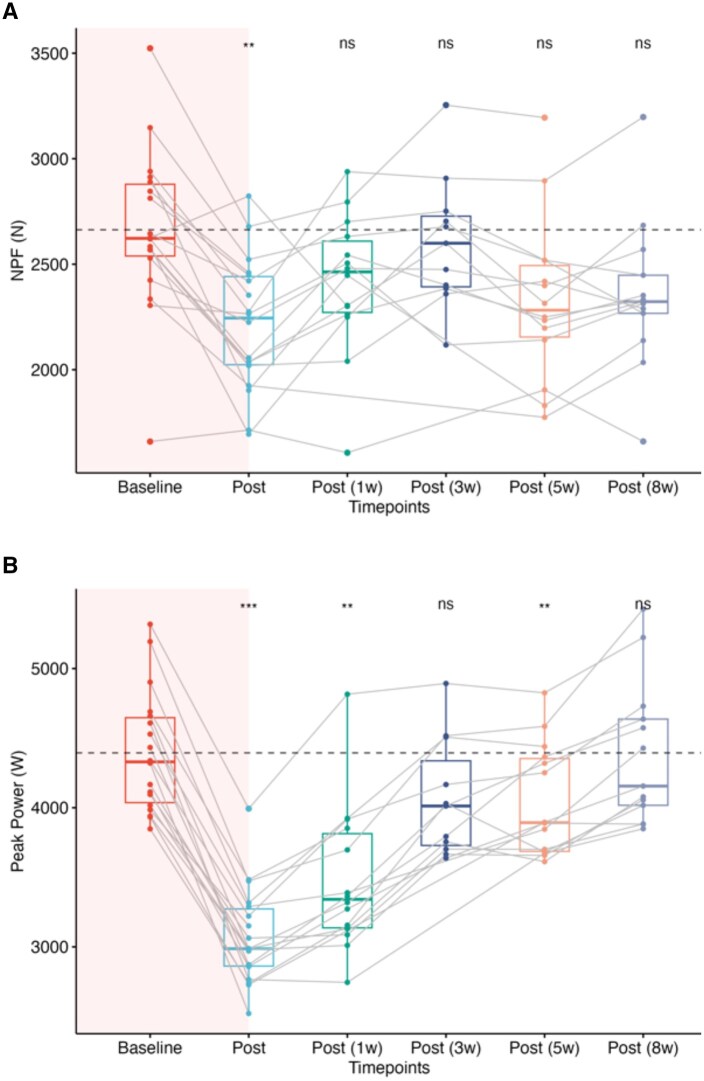
Box and whisker plots of changes in (A) IMTP net peak force (Newtons) and (B) CMJ peak power (watts) in response to the SF-SC and throughout 8 week follow-up. Red shading represents changes from pre to post SF-SC. Dots and connected lines represent each participant’s changes over time, and box and whiskers present group median and quartiles. Statistical differences represent differences compared to baseline from Wilcoxon rank test at level of significance: *P < .05, **P < .01, ***P < .001, ****P < .0001. Abbreviations: CMJ, counter movement jump; IMTP, isometric midthigh pull; NPF, net peak force; ns, nonsignificant; SF-SC, Special Forces Selection Course.

Peak force was significantly reduced by 16 ± 11% (*P* < .01, ES = 0.83, 95% CI [0.66-0.88]) (**Figure 1**), and PF/LBM was significantly reduced by 13 ± 13% (*P* < .05, ES = 0.76, 95% CI [0.54-0.88]) in response to the SF-SC (**Table 1**). Although no other IMTP variable demonstrated statistically significant changes in response to the SF-SC, the effect sizes for decreases in force at 200 ms (ES = 0.52, 95% CI [0.10-0.81]) and 250 ms (ES = 0.70, 95% CI [0.39-0.88]) were large. Similarly, peak RFD (ES = 0.51, 95% CI [0.13-0.82]), RFD 0-200 ms (ES = 0.53, 95% CI [0.12-0.82]), and RFD 0-250 ms (ES = 0.69, 95% CI [0.34-0.88]) also showed large effect sizes. All measured IMTP variables returned to baseline levels by 1 week post-course.

#### Countermovement jump (CMJ)

Jump height was reduced by 28 ± 9% (*P* < .001, ES = 0.88, 95% CI [0.88-0.88]) in response to the SF-SC. Peak power (**Figure 1**) and mean power were reduced by 30 ± 5.6% (*P* < .001, ES = 0.88, 95% CI [0.88-0.88]) and 29 ± 7.5% (*P* < .001, ES = 0.88, 95% CI [0.88-0.88]), respectively. Concentric peak force was also reduced by 26 ± 10% (*P* < .001, ES = 0.88, 95% CI [0.88-0.88]), braking peak force by 28 ± 26.3% (*P* < .01, ES = 0.86, 95% CI [0.76-0.88]), and FT:CT by 26 ± 10.8% (*P* < .001, ES = 0.88, 95% CI [0.88-0.88]). After 1 week of recovery, FT:CT, concentric peak force, eccentric RFD, braking RFD and braking peak force were recovered to baseline, whereas jump height (28 ± 14.3%), concentric Imp (16 ± 8.6%), mean power (21.3 ± 9.5%) and peak power (23 ± 8%) remained significantly reduced (*P* < .01, ES = 0.88, 95% CI [0.88-0.89]) (**Table 1**). All CMJ variables were recovered to baseline levels at 3 weeks post-course.

### Body Composition

The SF-SC resulted in large significant decreases in body mass (8.2 ± 2.3%, *P* < .01, ES = 0.88, 95% CI [0.88-0.88]), lean mass (4.3 ± 2.4%, *P* < .0001, ES = 0.88, 95% CI [0.88-0.88]) and fat mass (36 ± 6.1%, *P* < .0001, ES = 0.88, 95% CI [0.88-0.88]), which took between 1 and 3 weeks to recover.^14^ At 5 weeks post-course, body mass (ES = 0.69, 95% CI [0.41-0.88]) and lean mass (ES = 0.72, 95% CI [0.44-0.88]) were significantly higher (*P* < .05) compared to baseline levels; however, these differences were non-significant at 8 weeks post-course (**Table 2**).

### Hormones

There were large significant (*P* < .001, ES = 0.88, 95% CI [0.88-0.88]) decreases in TT (−86.3 ± 7.8%), FT (−92.6 ± 4.6) and T:C ratio (−91 ± 5.4%), and a large significant (*P* < .001, ES = 0.88, 95% CI [0.88-0.88]) increase in Cort (74.9 ± 51.5%) and SHBG (131.1 ± 59.3%) in response to the SF-SC.^14^ All hormones except FT had recovered to baseline levels by 1 week post-course, while FT was recovered by 3 weeks post-course (**Table 3**). Total testosterone again demonstrated a large reduction (17.3 ± 20.7%, *P* < .05, ES = 0.67, 95% CI [0.34-0.85]) at 5 weeks post-course; however, this hormone returned to baseline levels at 8 weeks post-course.

### Linear Mixed Effects Models

The LMEs demonstrated that TT, FT, and T:C ratio all had a significant effect on jump height, peak power, peak force and peak RFD, with the standardized beta coefficients indicating that increases in anabolic hormone levels were associated with positive changes in performance outcomes during both the IMTP and CMJ. Furthermore, both body mass and lean mass exhibited significant positive effects on peak power and peak force, while not displaying similar effects on jump height or peak RFD. For jump height, the model of best fit included FT alone (β = .47, 95% CI [0.31-0.63], *P* < .001). The most predictive model of peak power included body mass (β = .36, 95% CI [0.19-0.54], *P* < .001) and FT (β = .44, 95% CI [0.31-0.57], *P* < .001). For peak force, the optimal model included body mass (β = .29, 95% CI [0.11-0.47], *P* = .002) and TT (β = .29, 95% CI [0.15-0.42], *P* < .001). Conversely, for peak RFD, the model with the lowest AIC solely incorporated the TT term (β = .31, 95% CI [0.14-0.47], *P* < .001). LME results describing each model are presented in **Tables S1-S6**.

## DISCUSSION

The primary purpose of this study was to examine the effects of the SF-SC on physical performance variables (i.e., strength and power) and the time course of their recovery. Maximal strength significantly declined by 16% but recovered within 1 week, whereas peak power output showed a larger decrement (30%) and required up to 3 weeks to return to baseline. When physical performance was considered in conjunction with the observed changes in body composition and hormones, recovery of strength occurred simultaneously with recovery of body mass, lean mass, TT, and Cort, whereas recovery of power was delayed. The secondary aim of this study was to investigate the effects of lean mass, body mass, TT, FT, and T:C, on strength and power-generating capacities. Linear mixed effects modeling demonstrated that testosterone and body mass were the most important factors affecting jump height, peak power, peak force, and peak RFD, with body mass positively associated with peak power and peak force, FT positively associated with jump height and peak power, and TT positively associated with peak force and peak RFD.

A previous investigation of the Australian SF-SC reported significant (*P* < .05) performance decrements in response to the course, including IMTP gross peak force (∼−28%), gross PF/BM (∼−12%), CMJ jump height (∼−16%), and CMJ concentric peak force (∼−9%).^5^ In contrast to the previous study, we observed a relatively larger decrease in CMJ jump height (−28%, *P* < .001), and concentric peak force (−26%, *P* < .001), a relatively smaller decrease in IMTP net peak force (−16%, *P* < .01), and a smaller decrease in peak force relative to body mass (−5.0 ± 9.8%, *P* > .05). The reasons for the differences between the present and former study are unclear; however, it may be due to differences in testing methods, equipment used, and force-time curve analyses (i.e., net vs. gross) performed. The decreases in strength and power observed in the present study were, however, similar to changes observed in other studies that have examined strenuous military training.^1^^,^^3^^,^^25^ For example, a 20% decrease in maximal isometric lower body strength (i.e., leg press peak force) and 28% decrease in CMJ jump height have been reported following an arduous 7-day period within the Norwegian SF selection course.^1^ Similarly, a 20% decrease in strength (i.e., simulated power clean 1RM), and a 21% decrease in maximal power output during the vertical jump have been reported after an 8 week U.S. Army Ranger Training Course.^3^^,^^25^

The energy deficit often associated with military training is at least partly responsible for the observed decrements in physical performance.^26^ A meta regression exploring relationships between energy deficit, body composition, and physical performance reported that military activities producing a total energy deficit of 39,243-59,377 kcal are expect to result in body mass declines of >7.7%, and moderate (7%) to large (10%) decreases in physical performance.^26^ Therefore, according to the substantial energy deficit and the 8.2 ± 2.3% decrease in body mass observed in response to the SF-SC, the large decrease in physical performance following the SF-SC was expected. The authors of the meta regression recommended reducing the total energy deficit to curtail the loss of body mass, minimize losses of physical performance, and thereby maintain operational readiness,^26^ which is supported by other studies.^27–29^ Results from the LMEs demonstrated that body mass, TT and FT were the most important variables contributing to physical performance in the present study. As decreases in body mass, lean mass, TT and FT are all potential outcomes of energy deficit, our data support the concept that reducing the overall energy deficit may attenuate decreases in physical performance.

In addition to energy deficit and the associated loss of body mass and lean mass, other mechanisms have been proposed for decreases in physical performance following military training.^1^^,^^7^^,^^30^ It has been suggested that the decrease in testosterone may be related to the decrease in explosive power, due to the modulating effect of anabolic hormones on intracellular calcium in skeletal muscle.^7^ Alternatively, muscle damage has been proposed to be a primary cause of decreased physical performance during the Norwegian SF-SC, due to performance deficits persisting beyond the recovery of hormones and body composition, and findings of highly elevated (700% ± 200%) creatine kinase.^1^ Similarly, muscle damage, soreness and fatigue were attributed for the decrease in physical performance observed after a 7-day winter military training activity, as peak power was reduced despite only a modest (∼2%) change in body mass, whereas subjective ratings of pain and biomarkers of muscle damage were elevated.^30^ In support of this hypothesis, increased pro-inflammatory cytokines such as IL-6 and TNF-a, which are reported to be positively associated with exercise-induced muscle damage and symptoms of overtraining and fatigue,^31^^,^^32^ have been demonstrated in response to intense military training.^33–35^ Given the multi-factor stressors involved in the SF-SC, the large catabolic hormonal disruption, and the persistence of performance deficit beyond recovery of body mass and lean mass, it is likely that a combination of the abovementioned mechanisms contributed to the performance changes observed in the present study.

Despite large disruptions to physical performance, body composition and hormonal status, all strength (IMTP) variables tested were recovered 1 week following the SF-SC, while all power (CMJ) variables did not recover until 3 weeks post-course. This is noteworthy, as previous studies have linked vertical jump performance to operationally relevant tasks such as 30-meter rushes from prone (*r* = −0.72, *P* < .01), casualty drag (*r* = −0.64, *P* < .01), and a timed obstacle course under load (*r* = −0.62, *P* < .01).^11^^,^^36^ Moreover, power has been reported to be negatively correlated (*r* = −0.83, *P* < .001) with susceptibility to enemy fire, and together with critical velocity predicted 74% of the variance in susceptibility to enemy fire.^12^ Therefore, prolonged decreases in power following arduous military training may have undesirable implications for soldiers’ functional output and safety during subsequent training or operations. It is evident that allocating sufficient time and resources for targeted recovery strategies following training such as the SF-SC, is crucial.

A novel aspect of this study was the use of force plate methods for the assessment of strength and power, with a detailed analysis of the force-time curve for the IMTP and CMJ. Force plate testing has been recommended as a valuable and relevant method for the assessment of performance adaptations and neuromuscular fatigue within military populations;^37^^,^^38^ however, to date, there have been only limited studies using these methods before and after arduous military training. The IMTP analysis demonstrated significant declines in peak force and PF/LBM in response to the SF-SC. Other variables such as force at 250 ms and RFD 0-250 ms demonstrated large decreases (ES = 0.69–0.70) in response to the course; however, these differences were not statistically significant. It is possible that the relatively small sample size limited the ability for the changes in these variables to reach significance. All CMJ variables demonstrated large (∼26%-30%, *P* < .05, ES = 0.88, 95% CI [0.76-0.88]) decreases in response to the SF-SC, with jump height and power variables taking 3 weeks to recover to baseline levels. The CMJ is commonly utilized for monitoring neuromuscular fatigue in sporting populations,^39^ and has been recommended for use for monitoring performance and fatigue in military populations.^2^^,^^38^ Our data support the use of the CMJ as a sensitive method for monitoring acute and prolonged fatigue following strenuous military training.

Although the current study provides valuable information, there are several limitations that must be noted. The sample size available for analysis, especially throughout the recovery period, was small due to course attrition and changing availability of participants. This limitation, which is common in similar military-based studies,^6^^,^^7^^,^^33^ may have reduced the statistical power to detect additional changes in measured variables. Another limitation is that there were no female participants, even though the SF-SC is open to female candidates. It is plausible that the magnitude of changes and time-course of recovery may be different for female participants due to sex specific differences in body composition and hormones; therefore, future research should investigate the consequences of these types of courses in female soldiers. Finally, we did not assess all aspects of performance relevant to military populations. Measures assessing aerobic and anaerobic capacity, speed, agility, or muscular endurance may have demonstrated different performance decrements and recovery timeframes, and therefore should be considered in future research.

## CONCLUSIONS

The SF-SC is a highly strenuous period of military training. The combined stressors of the course resulted in substantial decreases in strength and power, in association with losses of body mass and lean mass, and hormonal disruption, which were not fully recovered until 3 weeks following the course. The results of this study can be used to inform training programs, ensuring adequate time and appropriate recovery interventions are applied following strenuous military training, in order to reduce risk of injury while performance is compromised.

## Supplementary Material

usaf541_Supplementary_Data

## Data Availability

Data generated or analyzed during this study are not publicly available due to the classified nature of the course and participants.

## INSTITUTIONAL CLEARANCE

Institutional clearance approved by Special Operations Command HQ.
