# Supplementary material for: Neuromuscular Performance Changes in Response to the Australian Special Forces Selection Course
Source: Mil Med. 2026 Apr 17;191(5-6):e1122–30. doi: 10.1093/milmed/usaf541 (PMC13143296; doi:10.1093/milmed/usaf541)
Supplement: usaf541_Supplementary_Data [file usaf541_supplementary_data.docx]

## Supplementary Tables

| **Table S1** LME model: Body mass and Free testosterone | | | | | | | | | | | | |
| --- | --- | --- | --- | --- | --- | --- | --- | --- | --- | --- | --- | --- |
| **Predictors** | **Jump Height** | | | **Peak Power** | | | **Peak Force** | | | **Peak RFD** | | |
|  | **Estimates** | **CI** | **p** | **Estimates** | **CI** | **p** | **Estimates** | **CI** | **p** | **Estimates** | **CI** | **p** |
| Age baseline | -0.17 | -0.43 – 0.09 | 0.193 | -0.18 | -0.40 – 0.05 | 0.121 | 0.01 | -0.29 – 0.31 | 0.971 | 0.15 | -0.12 – 0.43 | 0.264 |
| Time | 0.16 | 0.02 – 0.30 | **0.026** | 0.18 | 0.07 – 0.29 | **0.002** | 0.1 | -0.01 – 0.22 | 0.079 | 0.16 | 0.02 – 0.30 | **0.023** |
| Body mass | -0.04 | -0.26 – 0.17 | 0.676 | 0.36 | 0.19 – 0.54 | **<0.001** | 0.26 | 0.06 – 0.46 | **0.01** | -0.03 | -0.24 – 0.19 | 0.795 |
| Free testosterone | 0.47 | 0.31 – 0.63 | **<0.001** | 0.44 | 0.31 – 0.57 | **<0.001** | 0.23 | 0.09 – 0.36 | **0.001** | 0.25 | 0.09 – 0.41 | **0.002** |
| **Marginal R2 / Conditional R2** | 0.291 / 0.540 | | | 0.495 / 0.693 | | | 0.196 / 0.642 | | | 0.151 / 0.474 | | |
| **Note:** LME = linear mixed effects, RFD= rate of force development, CI = confidence interval. Estimates = estimated change in dependent variable for one unit change in predictor variable. Units of predictor variables have been scaled for consistency of comparison | | | | | | | | | | | | |

| **Table S2** LME model: Body mass and Total testosterone | | | | | | | | | | | | |
| --- | --- | --- | --- | --- | --- | --- | --- | --- | --- | --- | --- | --- |
| **Predictors** | **Jump Height** | | | **Peak Power** | | | **Peak Force** | | | **Peak RFD** | | |
|  | **Estimates** | **CI** | **p** | **Estimates** | **CI** | **p** | **Estimates** | **CI** | **p** | **Estimates** | **CI** | **p** |
| Age baseline | -0.25 | -0.55 – 0.04 | 0.085 | -0.25 | -0.48 – -0.02 | **0.035** | -0.05 | -0.34 – 0.23 | 0.706 | 0.09 | -0.20 – 0.38 | 0.545 |
| Time | 0.21 | 0.06 – 0.35 | **0.005** | 0.23 | 0.11 – 0.35 | **<0.001** | 0.09 | -0.02 – 0.20 | 0.124 | 0.15 | 0.01 – 0.28 | **0.033** |
| Body mass | 0.11 | -0.10 – 0.32 | 0.3 | 0.51 | 0.33 – 0.68 | **<0.001** | 0.29 | 0.11 – 0.47 | **0.002** | 0 | -0.20 – 0.20 | 0.984 |
| Total testosterone | 0.37 | 0.20 – 0.54 | **<0.001** | 0.33 | 0.19 – 0.47 | **<0.001** | 0.29 | 0.15 – 0.42 | **<0.001** | 0.31 | 0.14 – 0.47 | **<0.001** |
| **Marginal R2 / Conditional R2** | 0.239 / 0.536 | | | 0.473 / 0.667 | | | 0.238 / 0.642 | | | 0.165 / 0.525 | | |
| **Note:** LME = linear mixed effects, RFD= rate of force development, CI = confidence interval. Estimates = estimated change in dependent variable for one unit change in predictor variable. Units of predictor variables have been scaled for consistency of comparison | | | | | | | | | | | | |

| **Table S3** LME model: Body mass and Testosterone: Cortisol ratio | | | | | | | | | | | | |
| --- | --- | --- | --- | --- | --- | --- | --- | --- | --- | --- | --- | --- |
| **Predictors** | **Jump Height** | | | **Peak Power** | | | **Peak Force** | | | **Peak RFD** | | |
|  | **Estimates** | **CI** | **p** | **Estimates** | **CI** | **p** | **Estimates** | **CI** | **p** | **Estimates** | **CI** | **p** |
| Age baseline | -0.21 | -0.50 – 0.07 | 0.139 | -0.21 | -0.42 – -0.00 | **0.047** | -0.02 | -0.31 – 0.27 | 0.897 | 0.11 | -0.18 – 0.40 | 0.439 |
| Time | 0.32 | 0.18 – 0.46 | **<0.001** | 0.34 | 0.22 – 0.46 | **<0.001** | 0.18 | 0.07 – 0.29 | **0.001** | 0.23 | 0.10 – 0.36 | **<0.001** |
| Body mass | 0.2 | -0.02 – 0.41 | 0.071 | 0.59 | 0.42 – 0.76 | **<0.001** | 0.4 | 0.22 – 0.58 | **<0.001** | 0.07 | -0.13 – 0.27 | 0.495 |
| T:C | 0.15 | -0.01 – 0.31 | 0.063 | 0.11 | -0.03 – 0.24 | 0.127 | 0.07 | -0.05 – 0.20 | 0.251 | 0.18 | 0.03 – 0.33 | **0.017** |
| **Marginal R2 / Conditional R2** | 0.181 / 0.460 | | | 0.458 / 0.590 | | | 0.217 / 0.617 | | | 0.132 / 0.488 | | |
| **Note:** LME = linear mixed effects, RFD= rate of force development, T:C=testosterone: cortisol ratio. CI = confidence interval. Estimates = estimated change in dependent variable for one unit change in predictor variable. Units of predictor variables have been scaled for consistency of comparison | | | | | | | | | | | | |

| **Table S4** LME model: Lean mass and Free testosterone | | | | | | | | | | | | |
| --- | --- | --- | --- | --- | --- | --- | --- | --- | --- | --- | --- | --- |
| **Predictors** | **Jump Height** | | | **Peak Power** | | | **Peak Force** | | | **Peak RFD** | | |
|  | **Estimates** | **CI** | **p** | **Estimates** | **CI** | **p** | **Estimates** | **CI** | **p** | **Estimates** | **CI** | **p** |
| Age baseline | -0.16 | -0.42 – 0.10 | 0.217 | -0.17 | -0.40 – 0.07 | 0.157 | 0.02 | -0.30 – 0.33 | 0.921 | 0.17 | -0.11 – 0.45 | 0.219 |
| Time | 0.15 | 0.01 – 0.29 | **0.036** | 0.18 | 0.06 – 0.30 | **0.003** | 0.1 | -0.02 – 0.22 | 0.11 | 0.14 | 0.00 – 0.28 | **0.048** |
| Lean mass | -0.1 | -0.34 – 0.14 | 0.421 | 0.32 | 0.11 – 0.53 | **0.003** | 0.19 | -0.06 – 0.45 | 0.127 | -0.17 | -0.43 – 0.08 | 0.177 |
| Free testosterone | 0.48 | 0.33 – 0.63 | **<0.001** | 0.49 | 0.36 – 0.62 | **<0.001** | 0.27 | 0.14 – 0.41 | **<0.001** | 0.28 | 0.13 – 0.43 | **<0.001** |
| **Marginal R2 / Conditional R2** | 0.295 / 0.544 | | | 0.471 / 0.687 | | | 0.168 / 0.646 | | | 0.170 / 0.505 | | |
| **Note:** LME = linear mixed effects, RFD= rate of force development, CI = confidence interval. Estimates = estimated change in dependent variable for one unit change in predictor variable. Units of predictor variables have been scaled for consistency of comparison | | | | | | | | | | | | |

| **Table S5** LME model: Lean mass and Total testosterone | | | | | | | | | | | | |
| --- | --- | --- | --- | --- | --- | --- | --- | --- | --- | --- | --- | --- |
| **Predictors** | **Jump Height** | | | **Peak Power** | | | **Peak Force** | | | **Peak RFD** | | |
|  | **Estimates** | **CI** | **p** | **Estimates** | **CI** | **p** | **Estimates** | **CI** | **p** | **Estimates** | **CI** | **p** |
| Age baseline | -0.25 | -0.54 – 0.04 | 0.085 | -0.26 | -0.51 – -0.01 | **0.039** | -0.06 | -0.35 – 0.24 | 0.699 | 0.1 | -0.20 – 0.40 | 0.498 |
| Time | 0.21 | 0.06 – 0.35 | **0.006** | 0.24 | 0.11 – 0.36 | **<0.001** | 0.09 | -0.03 – 0.21 | 0.129 | 0.13 | -0.00 – 0.27 | 0.054 |
| Lean mass | 0.08 | -0.16 – 0.33 | 0.508 | 0.51 | 0.30 – 0.72 | **<0.001** | 0.27 | 0.05 – 0.49 | **0.018** | -0.12 | -0.36 – 0.13 | 0.346 |
| Total testosterone | 0.39 | 0.22 – 0.55 | **<0.001** | 0.39 | 0.25 – 0.54 | **<0.001** | 0.32 | 0.19 – 0.46 | **<0.001** | 0.33 | 0.17 – 0.48 | **<0.001** |
| **Marginal R2 / Conditional R2** | 0.237 / 0.529 | | | 0.457 / 0.667 | | | 0.223 / 0.643 | | | 0.176 / 0.548 | | |
| **Note:** LME = linear mixed effects, RFD= rate of force development, CI = confidence interval. Estimates = estimated change in dependent variable for one unit change in predictor variable. Units of predictor variables have been scaled for consistency of comparison | | | | | | | | | | | | |

| **Table S6** LME model: Lean mass and Testosterone: Cortisol ratio | | | | | | | | | | | | |
| --- | --- | --- | --- | --- | --- | --- | --- | --- | --- | --- | --- | --- |
| **Predictors** | **Jump Height** | | | **Peak Power** | | | **Peak Force** | | | **Peak RFD** | | |
|  | **Estimates** | **CI** | **p** | **Estimates** | **CI** | **p** | **Estimates** | **CI** | **p** | **Estimates** | **CI** | **p** |
| Age baseline | -0.2 | -0.48 – 0.07 | 0.141 | -0.21 | -0.43 – 0.02 | 0.072 | -0.02 | -0.33 – 0.29 | 0.909 | 0.13 | -0.16 – 0.42 | 0.375 |
| Time | 0.32 | 0.18 – 0.47 | **<0.001** | 0.36 | 0.23 – 0.49 | **<0.001** | 0.2 | 0.08 – 0.31 | **0.001** | 0.23 | 0.10 – 0.36 | **0.001** |
| Lean mass | 0.12 | -0.12 – 0.36 | 0.331 | 0.56 | 0.35 – 0.76 | **<0.001** | 0.38 | 0.15 – 0.62 | **0.002** | -0.06 | -0.31 – 0.18 | 0.612 |
| T:C | 0.17 | 0.01 – 0.33 | **0.036** | 0.15 | 0.00 – 0.29 | **0.044** | 0.1 | -0.03 – 0.23 | 0.137 | 0.21 | 0.06 – 0.35 | **0.006** |
| **Marginal R2 / Conditional R2** | 0.170 / 0.422 | | | 0.415 / 0.569 | | | 0.196 / 0.620 | | | 0.132 / 0.492 | | |
| **Note:** LME = linear mixed effects, RFD= rate of force development, T:C = testosterone: cortisol ratio, CI = confidence interval. Estimates = estimated change in dependent variable for one unit change in predictor variable. Units of predictor variables have been scaled for consistency of comparison | | | | | | | | | | | | |
